# Structural and mutational analyses of psychrophilic and mesophilic adenylate kinases highlight the role of hydrophobic interactions in protein thermal stability

**DOI:** 10.1063/1.5089707

**Published:** 2019-03-25

**Authors:** Sojin Moon, Junhyung Kim, Jasung Koo, Euiyoung Bae

**Affiliations:** 1 Department of Agricultural Biotechnology, Seoul National University, Seoul 08826, South Korea; 2 Research Institute of Agriculture and Life Sciences, Seoul National University, Seoul 08826, South Korea

## Abstract

Protein thermal stability is an important field since thermally stable proteins are desirable in many academic and industrial settings. Information on protein thermal stabilization can be obtained by comparing homologous proteins from organisms living at distinct temperatures. Here, we report structural and mutational analyses of adenylate kinases (AKs) from psychrophilic *Bacillus globisporus* (AKp) and mesophilic *Bacillus subtilis* (AKm). Sequence and structural comparison showed suboptimal hydrophobic packing around Thr26 in the CORE domain of AKp, which was replaced with an Ile residue in AKm. Mutations that improved hydrophobicity of the Thr residue increased the thermal stability of the psychrophilic AKp, and the largest stabilization was observed for a Thr-to-Ile substitution. Furthermore, a reverse Ile-to-Thr mutation in the mesophilic AKm significantly decreased thermal stability. We determined the crystal structures of mutant AKs to confirm the impact of the residue substitutions on the overall stability. Taken together, our results provide a structural basis for the stability difference between psychrophilic and mesophilic AK homologues and highlight the role of hydrophobic interactions in protein thermal stability.

## INTRODUCTION

Protein thermal stability is an important field since thermally stable proteins are advantageous in many industrial and laboratory settings.^1–6^ These proteins can maintain their native three-dimensional structures and remain functional at elevated temperatures, which allows for increased reaction rates, improved solubility, and reduced bacterial contamination in industrial processes. Thermal stability is also desirable for the manufacturing of protein-based pharmaceuticals. In the laboratory, thermally stable proteins are more convenient to store and handle and can be used in experimental procedures requiring high reaction temperatures such as thermophilic DNA polymerases in PCR.

Information on protein thermal stabilization can be obtained by comparing homologous proteins from organisms living at different temperatures. In numerous studies, psychrophilic and thermophilic proteins have been compared with their mesophilic homologues to identify the molecular basis for protein thermal stability.^5–10^ Changes in non-covalent intramolecular interactions such as electrostatic interactions, hydrogen bonds, and hydrophobic contacts have been proposed to account for the disparate structural stabilities between homologous proteins.^7–9,11–13^ This structural adjustment is considered to be the result of maintaining appropriate thermal motions required for physiological function at different environmental temperatures.^14–18^ However, different stabilizing interactions are used to different extents in different proteins, indicating that there is no unifying structural mechanism for protein thermal stabilization.

Adenylate kinase (AK) is a phosphotransferase enzyme catalyzing interconversion between ATP/AMP and two ADPs and is inhibited by P^1^,P^5^-di(adenosine 5′)-pentaphosphate (Ap_5_A), which mimics two adenylate substrates.^19,20^ AK has been used as a model enzyme in numerous biochemical and biophysical studies, including various thermal stability analyses.^21–27^ Structural studies have revealed three characteristic AK domains, namely, the CORE, AMP_bind_, and LID domains.^19,28–30^ We previously determined the crystal structures, thermal denaturation midpoints (T_m_), and temperature-dependent activities for AKs from a psychrophile, *Bacillus globisporus* (AKp), and a mesophile, *Bacillus subtilis* (AKm).^24,26,31^ The structural analysis revealed an overall decreased hydrophobic interaction in AKp compared to AKm.^26^ However, specific residue substitution(s) responsible for their stability difference have not been identified.

In the present study, a structural comparison of AKp and AKm revealed suboptimal hydrophobic packing in the CORE domain of the psychrophilic homologue, where a polar side chain makes contact with hydrophobic neighboring residues. Generation and T_m_ measurements of AK mutants confirmed that optimization of hydrophobic interactions in the CORE domain is important for overall thermal stability. We also determined the crystal structures of two AK mutants, which showed the largest thermal stability transition compared to wild-type (WT) AKs. Taken together, our results identified a single hydrophobic residue substitution that significantly affected overall thermal stability in AKs, highlighting the importance of hydrophobic packing in protein thermal stabilization.

## MATERIALS AND METHODS

### Cloning, expression, and purification

WT AKp and AKm genes were cloned into pET30a and pET11a vectors, respectively. Mutant genes were generated by site-directed mutagenesis using mismatched PCR primers. WT and mutant constructs were transformed into *Escherichia coli* BL21 (DE3). Proteins were expressed and purified by a two-step procedure involving affinity chromatography using Affi-Gel blue resin (Bio-Rad, USA) and size exclusion chromatography, as described previously.^24^ To purify the AKm I26T mutant, additional anion exchange chromatography was performed using a HiTrap Q Sepharose column (GE Healthcare) before the final size exclusion chromatography. The sample was loaded onto a column pre-equilibrated with anion exchange chromatography buffer (2 mM dithiothreitol, 20 mM Tris-HCl pH 8.0). The bound AKm I26T mutant was eluted by applying a linear gradient of NaCl (up to 1 M).

### Measurement of T_m_ values

T_m_ values of AKs were measured by circular dichroism (CD) spectroscopy, as described previously.^27^ CD traces at 220 nm were determined for 0.5 mg/ml of proteins in 10 mM potassium phosphate with pH 7.0. A Chirascan-plus CD Spectrometer (Applied Photophysics, UK) was used with a scanning rate of 1 °C/min. The thermal unfolding of AKs was irreversible under our experimental condition, and the scans were performed from low to high temperatures. CD data were analyzed based on the protocol developed by John and Weeks.^32^ Average values of three CD measurements at each temperature were processed to yield differential denaturation curves, which were fitted to non-linear regression parameters including T_m_ using a two-state transition model.

### Crystallization, data collection, and structure determination

The AKp T26I crystals were grown at 20 °C using the small-scale batch method^33^ from 20 mg/ml protein and 4 mM Ap_5_A in buffer (10 mM HEPES pH 7.0) mixed with an equal amount of reservoir solution [2.5 M ammonium sulfate, 1% (w/v) polyethylene glycol 1000, 0.1% (w/v) NaN_3_, 50 mM HEPES with pH 7.0]. Crystals were cryoprotected in reservoir solution supplemented with 20% (v/v) glycerol and flash-frozen in liquid nitrogen. AKm I26T crystals were grown at 20 °C by the hanging-drop method from 15 mg/ml protein and 4 mM Ap_5_A in buffer (10 mM HEPES with pH 7.0) mixed with an equal amount of reservoir solution [36% (w/v) polyethylene glycol 1500, 40 mM CaCl_2_, 50 mM CHES with pH 9.0]. Crystals were flash-frozen in liquid nitrogen without additional cryoprotectant.

Diffraction data were collected at 100 K at the beamlines AR-NW12A and BL-1A of the Photon Factory for AKp T26I and AKm I26T, respectively. Diffraction images were processed using HKL-2000.^34^ The structures of WT AKp and AKm (Protein Data Bank codes 1S3G and 1P3J, respectively) were used as starting models for molecular replacement phasing in Phaser.^35^ The structures were completed using alternate cycles of manual fitting in Coot^36^ and refinement in REFMAC5.^37^ The stereochemical quality of the final models was assessed using MolProbity.^38^

### Temperature-dependent activity assay

AK activity was measured at various temperatures in the direction of ATP formation as described previously.^27^ The enzymatic reaction was started with the addition of AK (1.1 ng/ml final concentration) to the reaction mixture (2.5 mM ADP, 1 mM glucose, 0.4 mM NADP^+^, 100 mM KCl, 2 mM MgCl_2_, 50 mM HEPES with pH 7.4). After incubation at the indicated temperatures for 5 min, the reaction was stopped by adding 0.5 mM Ap_5_A. The amount of ATP produced by the reaction was determined based on ATP-dependent NADP^+^ reduction to NADPH using hexokinase and glucose-6-phosphate dehydrogenase at room temperature.

### Accession numbers

The atomic coordinates and structure factors for AKp T26I and AKm I26T mutants were deposited in the Protein Data Bank^39^ under Accession Nos. 5X6J and 5X6I, respectively.

## RESULTS

### Suboptimal hydrophobic packing was identified in the CORE domain of the psychrophilic AKp

**FIG. 1.**
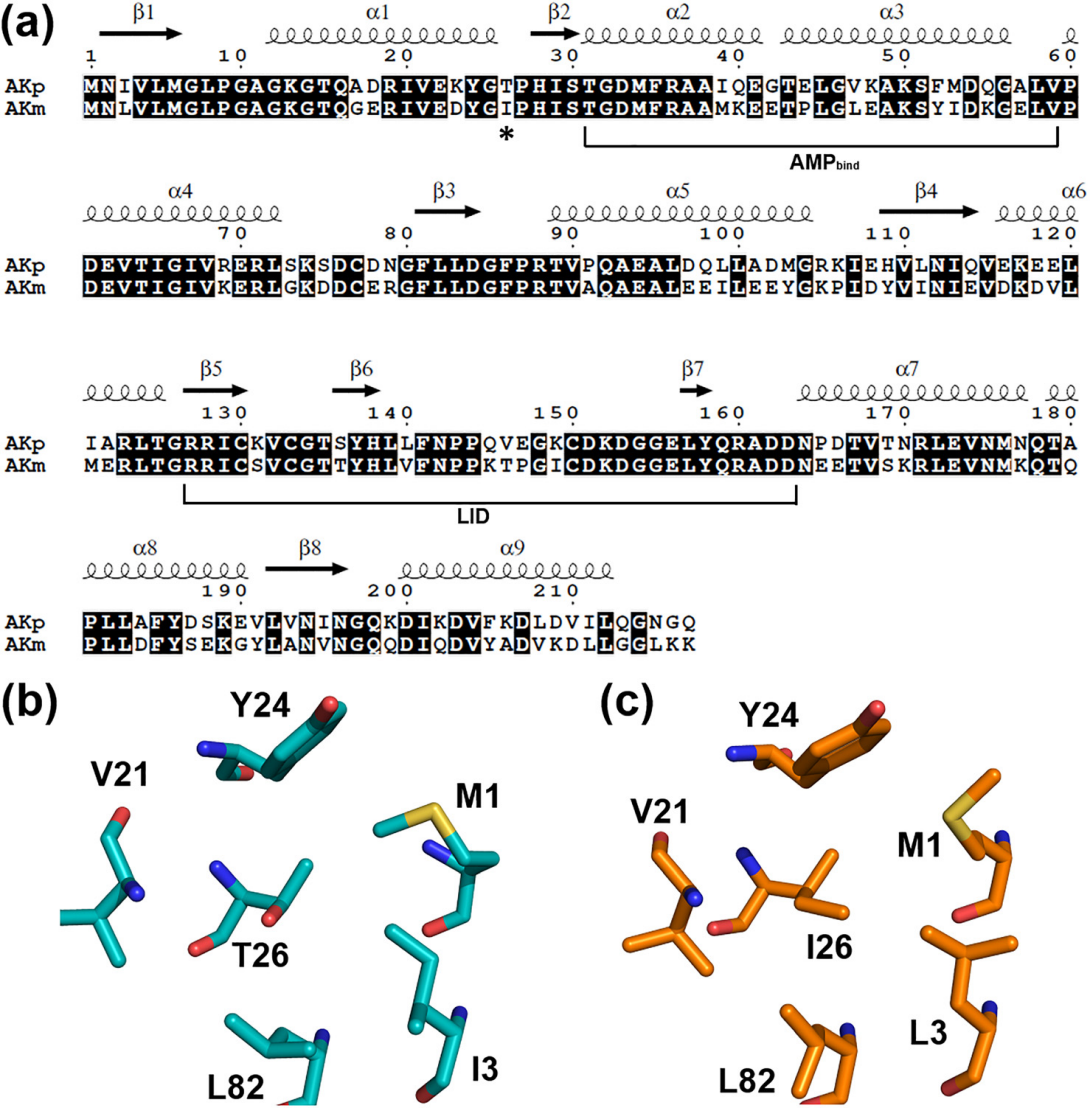
Sequence and structure of WT psychrophilic and mesophilic AKs. (a) Sequence alignment of AKp from psychrophilic *B. globisporus* and AKm from mesophilic *B. subtilis*. Secondary structural elements are indicated based on AKp. The position of the mutation (residue 26) is indicated by an asterisk. Residues of the AMP_bind_ (residues 31–59) and LID (residues 127–163) domains are also indicated. The CORE domain includes the remaining residues (residues 1–30, 60–126, and 164–217). (b) and (c) Close-up views of the hydrophobic environment around Thr26 in AKp (b) and Ile26 in AKm (c). The polar side chain of Thr26 in AKp cannot interact hydrophobically with adjacent non-polar residues, whereas Ile26 in AKm makes close hydrophobic contacts with other CORE residues.

In a previous structural comparison, mesophilic AKm showed more total apolar buried surface area than psychrophilic AKp,^26^ suggestive of increased overall hydrophobic interactions in AKm compared to AKp. In the present study, we attempted to identify specific residue substitution(s) that modify hydrophobic packing between the two AK homologues, resulting in a marked change in thermal stability. We focused on residues in AKm which were substituted to less hydrophobic amino acids in AKp. One such position was residue 26, located in the loop connecting α1 and β2 (Fig. 1). This residue belongs to the central CORE domain (residues 1–30, 60–126, and 164–217), which are known to be important for the overall structural stability of AKs.^22^

Mesophilic AKm contains Ile26 at this position, and its side chain is in close proximity (<5 Å) to other hydrophobic CORE residues, including Met1, Leu3, Val21, Tyr24, and Leu82 (Fig. 1). The psychrophilic AKp contains a more polar Thr26 instead of the Ile residue. However, most of the neighboring residues are conserved between AKm and AKp; the exception is Leu3 in AKm, which is substituted to Ile3 in AKp, indicating that the hydrophobic environment around residue 26 is very similar in the two AKs (Fig. 1). In the structure-based sequence alignment (Fig. S1), we found that other AKs also contain hydrophobic residues in the positions equivalent to Thr26 and the five neighboring residues in AKp. These observations strongly suggest that Thr26 in AKp disrupts hydrophobic packing in the CORE domain and likely decreases its thermal stability compared to other AKs including AKm. Notably, a thermally stable AK homologue from a thermophilic bacterium *Geobacillus stearothermophilus* also has an Ile residue at this position (Fig. S1),^26^ supporting that Ile26 is important for optimal hydrophobic CORE packing and thermal stabilization of AKm.

### Mutations improving hydrophobic packing in the CORE domain increased the thermal stability of AKp

To further assess the role of residue 26 in the thermal stability of AKs, we generated mutants of AKm and AKp, which harbored a single amino acid substitution at position 26 and measured their T_m_ values using CD spectroscopy (Table I and Fig. S2). Mutation of Ile26 in the mesophilic AKm to Thr (as found in psychrophilic AKp) resulted in a 7.0 °C reduction in T_m_. Consistent with this, the Thr-to-Ile substitution at residue 26 significantly increased the thermal stability of psychrophilic AKp, as indicated by a T_m_ value of 8.1 °C higher than the WT protein.

**TABLE I.** T_m_ values of WT and mutant AKs.

AK	Mutation	T_m_ (°C)	ΔT_m_ (°C)^a^
AKp	WT	42.7^b^	0.0
	T26N	38.3	−4.4
	T26A	38.4	−4.3
	T26S	38.7	−4.0
	T26F	43.0	0.3
	T26Y	44.1	1.4
	T26V	48.3	5.6
	T26L	48.4	5.7
	T26I	50.8	8.1
AKm	WT	46.4^b^	0.0
	I26T	39.4	−7.0

^a^ Difference from the T_m_ of WT AK.

^b^ T_m_ values of WT AKp and AKm have previously been determined.^24,31^ They were measured by CD spectrometry under the identical experimental protocol used in the present study.

To confirm that the changes in thermal stability resulted from modifications of hydrophobic interactions, we measured the T_m_ values of several other AKp mutants in which Thr26 was mutated to various amino acid residues (Table I). When Thr26 of AKp was substituted to amino acids containing a small or polar side chain, the thermal stability was decreased compared to WT AKp. The T_m_ values of the T26N, T26A, and T26S mutants were ∼4 °C lower than that of WT protein. Replacement with amino acid residues containing large aromatic side chains did not significantly affect the thermal stability; the T_m_ values of T26F and T26Y mutants were similar to that of WT AKp. A significant increase in thermal stability was observed only when Thr26 was mutated to amino acids with non-polar, aliphatic side chains. T_m_ of T26V and T26L mutants increased by ∼6 °C, and the largest thermal stabilization (8.1 °C) was conferred by the T26I substitution.

**FIG. 2.**
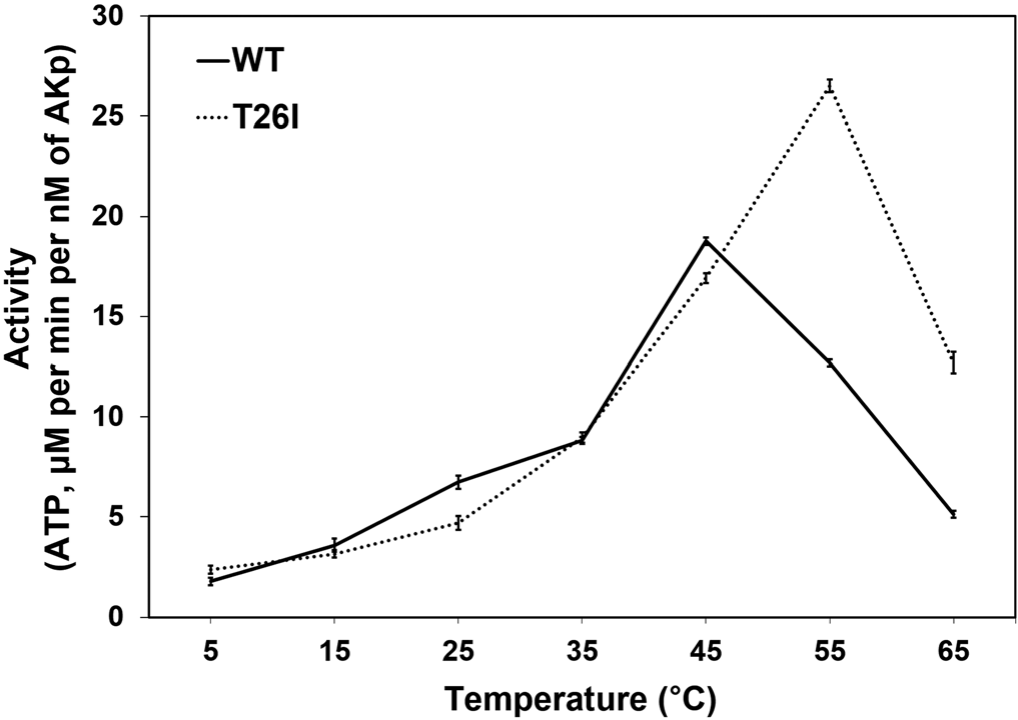
Temperature–activity profiles of WT AKp and the T26I mutant. Activity of the AKp T26I mutant was increased significantly compared to WT enzyme at high temperatures (55 and 65 °C), but their activities were very similar at low temperatures (5–45 °C). Data are represented as the means ± standard error.

To examine the effect of suboptimal hydrophobic CORE packing on the catalytic activity, we determined the temperature dependence of the activities of WT AKp and its T26I mutant (Fig. 2). Since the catalytic function was measured in the experimental condition different from that of the CD experiment, the results from the activity assays, such as the temperatures of maximum activities, were not directly compared with the T_m_ values. Temperature-activity profiles were analyzed relatively between WT and mutant AKs. The AKp T26I mutant showed increased catalytic activities at high temperatures (55 and 65 °C) compared to WT enzyme, and the temperature of maximum activity shifted from 45 °C to 55 °C by the mutation. This is consistent with the T_m_ measurement results (Table I) and can be explained by enhanced thermal stability of the T26I mutant compared to WT AKp. Interestingly, the Thr-to-Ile mutation did not significantly affect the catalytic activity of AKp at relatively low temperatures (5 °C to 45 °C). The activity assays of WT AKm and the I26T mutant also showed consistent results (Fig. S3). The I26T mutation in AKm significantly reduced catalytic activities at high temperatures (55 and 65 °C), whereas the activities at low temperatures (5 °C to 45 °C) were similar between the WT and mutant enzymes. These observations suggest that optimization of the hydrophobic CORE packing in AK is not directly associated with its functional dynamics, which involves motions of the other two AK domains, AMP_bind_ and LID (see Discussion).^28–30^

### Crystal structures of AK mutants confirmed the impact of hydrophobic CORE packing on the overall stability of AKs

**FIG. 3.**
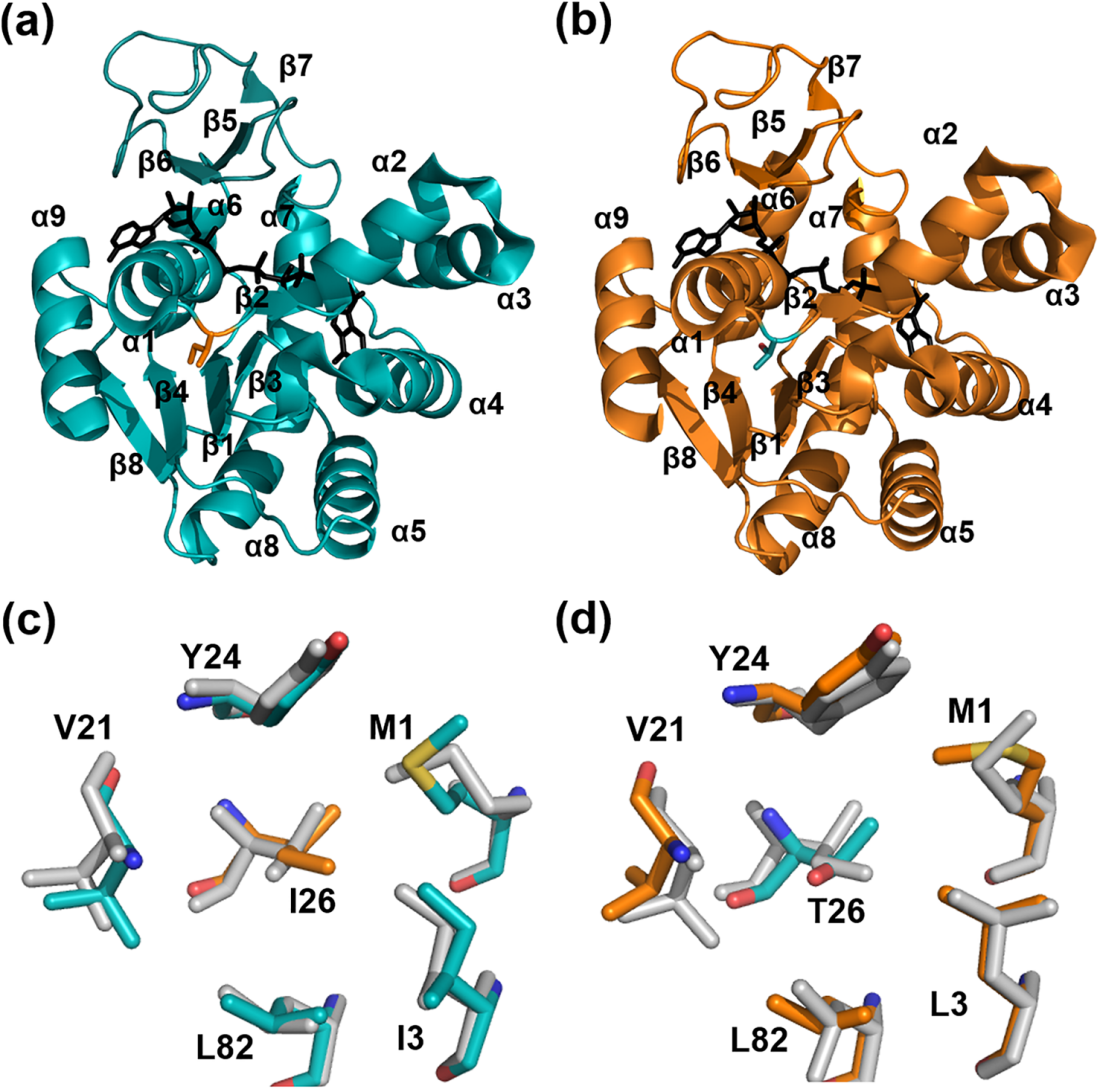
Crystal structures of the AK mutants, AKp T26I and AKm I26T. (a) and (b) Overall structures of AKp T26I (a) and AKm I26T (b). The co-crystallized inhibitor Ap_5_A molecule is shown in black. The mutated residues in each structure are highlighted in stick representations. (c) and (d) Effect of residue substitutions at position 26 on hydrophobic CORE packing of AKp T26I (c) and AKm I26T (d). The T26I substitution in AKp improves hydrophobic CORE packing. Ile26 (orange) of AKp T26I mutant interacts closely with other hydrophobic residues (cyan) in the CORE domain. The I26T mutation in AKm disrupts hydrophobic packing in the CORE domain. Thr26 in the AKm I26T mutant (cyan) cannot make hydrophobic contacts with other CORE residues (orange). The structures of WT AKp (c) and AKm (d) are also shown in grey for comparison.

We determined the crystal structures of AKp T26I and AKm I26T mutants to confirm the structural basis of the changes in thermal stability (Fig. 3). Data collection and refinement statistics are summarized in Table II. Analyses of the two mutant structures yielded further information supporting that mutations at residue 26 altered the hydrophobic intramolecular interactions in the CORE domain. In the structure of the T26I mutant of AKp, the side chain of Ile26 adopts a conformation almost identical to that of Ile26 in WT AKm and interacts closely with the neighboring hydrophobic CORE residues, as in the WT mesophilic homologue. For the WT and mutant AK structures, we calculated the apolar buried molecular surface area (Table III), which is considered as a measure of the degree of hydrophobic interactions. The AKp I26T mutant has increased the apolar buried surface area compared to WT, and almost half of the increase resulted from the six-residue cluster including Ile26 and the five neighboring residues. Consistently, the I26T substitution reduced the apolar buried surface area in AKm.

**TABLE II.** Data collection and refinement statistics.^a^

	AKp T26I	AKm I26T
Space group	P3_1_21	P2_1_2_1_2_1_
Unit cell parameters (Å)	a = 44.2, b = 55.2, c = 46.4,	a = 43.7, b = 44.2, c = 100.6
Wavelength (Å)	1.0000	0.9640
Data collection statistics		
Resolution range (Å)	50.00-2.10 (2.18-2.10)	50.00-2.00 (2.03-2.00)
Number of reflections	13 990 (1359)	13 718 (650)
Completeness (%)	99.8 (100.0)	99.9 (100.0)
R_merge_^b^	0.070 (0.508)	0.081 (0.580)
Redundancy	21.6 (21.9)	4.7 (4.7)
Mean I/σ	54.0 (8.8)	29.1 (2.9)
Refinement statistics		
Resolution range (Å)	50.00-2.10	50.00-2.00
R_cryst_^c^/R_free_^d^ (%)	19.7/27.6	18.3/25.9
RMSD bonds (Å)	0.016	0.015
RMSD angles (deg)	2.0	1.9
Average B factor (Å^2^)	49.3	43.4
Number of water molecules	89	104
Ramachandran favored (%)	97.2	98.1
Ramachandran allowed (%)	2.8	1.9

^a^ Values in parentheses are for the highest-resolution shell.

^b^ R_merge_ = Σ_h_Σ|I_i_(h) − ⟨I(h)⟩|/ Σ_h_Σ_i_I_i_(h), where I_i_(h) is the intensity of an individual measurement of the reflection and ⟨I(h)⟩ is the mean intensity of the reflection.

^c^ R_cryst_ = Σ_h_ǁF_obs_| − |F_calc_ǁ/Σ_h_|F_obs_|, where F_obs_ and F_calc_ are the observed and calculated structure factor amplitudes, respectively.

^d^ R_free_ was calculated as R_cryst_ using 5% of the randomly selected unique reflections that were omitted from structure refinement.

**TABLE III.** Apolar buried molecular surface area (Å^2^) of WT and mutant AK structures.^a^

		Residue 26	Six-residue cluster^b^	CORE domain^c^	All^d^
AKp	WT	21	218	3252	4699
	T26I	42	240	3283	4738
AKm	WT	39	241	3314	4846
	I26T	26	219	3295	4801

^a^ Calculated by using WHAT IF.^48^ Carbon and sulfur atoms were considered apolar.

^b^ Residues 1, 3, 21, 24, 26, and 82.

^c^ Residues 1–30, 60–126, and 164–212.

^d^ Residues 1–212.

The T26I mutant of AKp shows an average B-factor (50.0 Å^2^) similar to that of WT AKp (51.4 Å^2^) for all protein atoms (residues 1–213), whereas the average value for atoms of Ile26 (53.0 Å^2^) in the mutant is considerably smaller than that of Thr26 (60.7 Å^2^) in the WT structure, even though the side chain of Ile is longer than that of Thr. These observations suggest that the flexibility of residue 26 is decreased by Thr-to-Ile substitution in AKp, and the relative rigidity of the mutated Ile26 likely results from enhanced hydrophobic interactions with neighboring residues in the CORE domain. In contrast, in the crystal structure of the AKm I26T mutant, the polar side chain of the mutated Thr26 residue cannot have these hydrophobic contacts with other non-polar residues in the CORE domain. Taken together, our analyses suggest that modification of the hydrophobic packing in the CORE domain is important for the overall thermal stability of AK. Further investigation of protein dynamics using various techniques such as molecular dynamics simulation may help the validation of our suggestion.

## DISCUSSION

A comparison of homologous proteins adapted to distinct temperatures does not always guarantee the identification of critical structural features responsible for thermal stabilization in individual cases. Significant differences in thermal stability may be achieved by a combination of many subtle stabilizing factors. If homologous proteins are only distantly related, differences in the sequence and structure may or may not be relevant to their distinct operating temperatures, and it is difficult to discern changes responsible for temperature adaptation. Thus, it is important to compare homologues with similar amino acid sequences, but disparate temperature preferences.

In this study, we compared a pair of bacterial AKs from two species of the genus *Bacillus*. It was previously reported that the optimal growth temperature of the psychrophilic *B. globisporus* (5–10 °C) was significantly lower than that of the mesophilic *B. subtilis* (30–40 °C).^40^ Despite the disparate environmental temperatures of their source organisms, AKp and AKm exhibited ∼70% amino acid sequence identity. Hence, the closely related protein homologues used in this study facilitated the identification of specific structural features important for overall structural stability. Indeed, we identified a single residue substitution between the psychrophilic and mesophilic AKs that resulted in a significant change in thermal stability. Mutating only one residue in the CORE domain of AKp could significantly increase its T_m_ value by up to 8.1 °C (Table I).

Our structural and mutational analyses indicated that the significant thermal stabilization observed in the T26I mutant of AKp resulted from optimization of the hydrophobic CORE packing. Several other studies have shown that hydrophobic interactions in the CORE domain are important for the overall thermal stability of AKs. A structural comparison of mesophilic AKm and thermophilic *G. stearothermophilus* AK revealed a residue substitution (T179M) that could modify hydrophobic CORE packing.^26^ Introduction of the Thr-to-Met mutation at residue 179 increased the T_m_ of AKm by 2.5 °C.^24^ Enhanced hydrophobic interactions in the CORE domain were also found in thermally stabilized AKm mutants, which were generated by experimental evolution.^41^ The hydrophobicity-enhancing mutations include Q16L, T179I, and A193V, and the combination of Q16L and A193V resulted in a 13.8 °C increase in T_m_.^41^ A computational design and experimental characterization of 100 AKm variants showed that thermal stability could be conferred via repacking of the hydrophobic CORE.^42^ Interestingly, the positions of the stabilizing mutations found in these different studies are often identical, highlighting the importance of hydrophobic CORE packing in thermally stable AKs.

Residue substitutions that change the overall thermal stability by altering hydrophobic interactions in the CORE domain were also identified in eukaryotic AKs. In our recent study, AK from the Antarctic fish *Notothenia coriiceps* was compared with homologues from tropical fish, including *Danio rerio*.^27^ In the structural analysis, *N. coriiceps* AK showed suboptimal hydrophobic packing around three Val residues in the CORE domain, which was replaced with Ile residues in *D. rerio* AK.^27^ Mutations of these three Val residues to Ile collectively increased the T_m_ of Antarctic AK by 11.3 °C.^27^ Interestingly, the triple Val-to-Ile substitution also resulted in a significant reduction in catalytic activity at low temperatures,^27^ whereas in the present study, the activity of the T26I mutant of AKp was similar to that of WT protein from 5 °C to 45 °C (Fig. 2). This indicates that, unlike bacterial AKs, modification of the hydrophobic CORE packing in fish AKs can significantly affect the catalytic function.

The difference between bacterial and eukaryotic homologues is likely associated with structural variation of AK. Bacterial AKs have a long LID domain containing more than 30 residues,^20,26^ but the LID in fish AKs is essentially a short loop including less than 10 amino acid residues.^27^ Hence, it is possible that structural changes in the CORE domain can influence the dynamics of small eukaryotic LIDs, but not the large, independently folded bacterial domains. This is consistent with the results from previous studies on bacterial AKs, in which the catalytic activity was mediated by movements of the LID domain,^22,43–45^ whereas overall thermal stability was controlled by the structural integrity of the CORE domain.^22^ Notably, the Wolf-Watz group previously reported that bacterial AK domains fold in a noncooperative manner,^46^ and partial unfolding/refolding of the LID domain is important for catalysis.^47^

## SUPPLEMENTARY MATERIAL

See supplementary material for the additional analyses of the studied AKs (Figs. S1–S3).
